# Translational Simulation Improves Compliance with the NEAR4KIDS Airway Safety Bundle in a Single-center PICU

**DOI:** 10.1097/pq9.0000000000000409

**Published:** 2021-05-19

**Authors:** Nora Colman, Jordan W. Newman, Akira Nishisaki, Melinda Register, Scott E. Gillespie, Kiran B. Hebbar

**Affiliations:** From the * Department of Pediatrics, Division of Pediatric Critical Care, Children’s Healthcare of Atlanta, Atlanta, Ga.; † Department of Anesthesiology and Critical Care Medicine, The Children’s Hospital of Philadelphia, The University of Pennsylvania Perlman School of Medicine, Philadelphia, Pa.; ‡ Department of Pediatrics, Emory University School of Medicine, Atlanta, Ga.

## Abstract

The National Emergency Airway Registry for Children (NEAR4KIDS) Airway Safety Quality Improvement (QI) Bundle is a QI tool to improve the safety of tracheal intubations. The ability to achieve targeted compliance with bundle adherence is a challenge for centers due to competing QI initiatives, lack of interdisciplinary involvement, and time barriers. We applied translational simulations to identify safety and performance gaps contributing to poor compliance and remediate barriers by delivering simulation-based interventions.

**Methods:**

This was a single-center retrospective review following translational simulations to improve compliance with the NEAR4KIDS bundle . The simulation was implemented between March 2018 and December 2018. Bundle adherence was assessed 12 months before simulation and 9 months following simulation. Primary outcomes were compliance with the bundle and utilization of apneic oxygenation. The secondary outcome was the occurrence of adverse tracheal intubation-associated events.

**Results:**

Preintervention bundle compliance was 66%, and the application of apneic oxygenation was 27.9%. Following the simulation intervention, bundle compliance increased to 93.7% (*P* < 0.001) and adherence to apneic oxygenation increased to 77.9% (*P* < 0.001). There was no difference in the occurrence of tracheal intubation-associated events.

**Conclusions:**

Translational simulation was a safety tool that improved NEAR4KIDS bundle compliance and elucidated factors contributing to successful implementation. Through simulation, we optimized bundle customization through process improvement, fostered a culture of safety, and effectively engaged multidisciplinary teams in this quality initiative to improve adherence to best practices surrounding tracheal intubations.

## INTRODUCTION

Pediatric tracheal intubation (TI) is a high-risk procedure that requires complex coordination of care in a high-risk environment.^1^ The National Emergency Airway Registry for Children (NEAR4KIDS) is a multicenter, airway collaborative network created to improve pediatric intubations.^1–3^ In previously published NEAR4KIDS registry data, TI-associated events (TIAEs) occurred in 20% of intubations,^1–4^ resulting in increased length of mechanical ventilation, length of stay, and increased mortality.^5^ Clinician, patient, practice factors, poor team performance, ineffective communication, and improper intubation technique contributed to adverse events.^1,3^

The NEAR4KIDS Airway Safety Quality Improvement (QI) bundle checklist (further referred to as the “bundle”) is a QI tool that improves TI safety through standardization of practice, clinical decision support, and enhanced teamwork and communication through a shared mental model.^3^

Checklists are a powerful patient safety tool. However, the existence of a checklist does not ensure that it is applied effectively, successfully implemented as intended, or translates to behavioral or practice modification.^3,6^ In a study evaluating barriers to implementing the bundle, the inability to achieve the targeted >80% compliance was due to competing QI initiatives, lack of interdisciplinary involvement, education, buy-in, and time barriers.^6^

Fortunately, simulation has emerged as a safety strategy that offers potential solutions to address challenges that healthcare systems face in successfully implementing quality initiatives and improving the safety of care delivery.^3,7^ Translational simulation improves patient care and healthcare systems by identifying safety and performance gaps and delivering simulation-based interventions.^8^ Translational simulation fosters a culture of safety, improves team functioning, standardizes care processes and protocols, and provides a platform to devise potential solutions.^7^

Despite our center being part of the NEAR4KIDS registry since 2010, there was inconsistency in how and when teams utilized the bundle. Before our intervention, bundle and apneic oxygenation compliance was 66% and 27.9%, respectively, well below the target goal of >80%. These outcomes anecdotally correlated with intubations challenged by ineffective team performance and communication breakdown. This highlighted a critical opportunity to improve the quality and safety of care during intubations. Improving bundle adherence, therefore became the focus of our pediatric intensive care units (PICUs) annual simulation initiative. Before simulation, a video provided by the NEAR4KIDS network, highlighting the approach to bundle application, was reviewed with physicians and respiratory therapists (RTs) by our site leader as part of our core didactic curriculum in October 2016.^3^ Apneic oxygenation was added by NEAR4KIDS in 2016. Our center adapted the practice in January 2017, but no education had been initiated before the simulation.

We utilized a translational simulation approach to identify and remediate barriers that contributed to poor bundle compliance in our large academic pediatric ICU. We aimed to increase compliance with the bundle from 66% to 80%, apneic oxygenation from 27.9% to 80%, and reduce adverse TIAEs from 6.9% to 5.5% over 9 months.

## METHODS

### Intervention

This report is a single-center retrospective review of prospectively collected data following translational simulation to improve compliance with the bundle. The simulation was implemented in the PICU at Children’s Healthcare of Atlanta at Egleston (ECH) between March 2018 and December 2018. Bundle adherence was assessed 12 months before simulation (March 2017 to March 2018) and 9 months following simulation (January 2019 to October 2019). Primary outcomes were bundle compliance and utilization of apneic oxygenation during TIs. The secondary outcome was the occurrence of adverse TIAEs. The local NEAR4KIDS database provided bundle, apneic oxygenation compliance, and adverse TIAE rates. The project was determined to be nonhuman subject research by our Institutional Review Board.

### Participants and Setting

During a 10-month training period, we conducted 41 3-hour simulation workshops. Simulations took place in an in situ simulation laboratory room in the PICU, which consisted of a control station behind 1-way glass, a mounted monitor, and a high-fidelity Gaumard PediHal S3004 (1 year old) mannequin.

Interdisciplinary PICU staff, including nurses, RTs, PICU nurse practitioners, and PICU fellows, attended a single training as part of their annual competency requirements. The simulation was attended by 96% of nursing staff, 93% of RT staff, and 100% of the nurse practitioners and fellows. Each workshop consisted of 5–7 learners: 3–4 PICU nurses, 1–2 RTs, and 1 PICU fellow or PICU nurse practitioner. Of note, our training was a 3-hour workshop that included additional learning objectives beyond the scope of this article.

### Facilitation

Reflective Deliberate Practice, a debriefing approach blending Rapid Cycle Deliberate Practice (RCDP) and Traditional Reflective Debriefing (TRD) was used. RCDP provided just-in-time feedback and targeted coaching to learners, involved frequent pauses for feedback based on predetermined stops, and required learners to repeat skills until mastered. The facilitator observed the team for behaviors that either met or failed to meet learning objectives. If the team did not meet a learning objective, the entire team was paused and given immediate action-based feedback. Soft stop objectives necessitated feedback but not skill repetition.^9–13^ TRD occurred after the scenario and relied on learner reflection to explore strengths and weaknesses. Facilitators used debriefing techniques to assess the learner’s frame and close any performance gaps.^14^

A simulation technician, 1 PICU-based simulation educator, and 1 ICU physician with extensive training in delivering simulation, debriefing healthcare teams, and conducting RCDP training, were present at every workshop. Nurse and RT coaches provided discipline directed feedback to their respective learners during the facilitator directed RCDP pauses, whereas the primary facilitator (PICU physician) coached the physician learner.

RCDP coaches were senior-level nurses and RTs who held a leadership or educator role in our PICU. Coaches underwent formal training in the delivery of RCDP training by attending a 1-day class. Coaches and facilitators rehearsed scenarios. They refined and practiced the scenarios with members of the ICU leadership team.

### Simulation Event

The simulation included a single scenario with one intubation sequence. RCDP training (40 min) and TRD (40 min) coached learners in the appropriate timing, use, and application of the bundle. Each scenario was preprogrammed with time-sensitive triggers to prompt scenario progression. A detailed script with triggers and learning objectives divided into hard and soft stops was used during each workshop to standardize the training and minimize learner experience variation. The bundle checklist form was prepopulated for each workshop to maintain consistency. Learning objectives are in Table 1.

**Table 1.** Simulation Learning Objectives

Scenario Summary: 10-mo-old, ex 30-week infant with hypoxic respiratory failure secondary to bronchiolitis. Patient becomes hypoxic and bradycardic due to ETT dislodged requiring reintubation. Patient is difficult to mask requiring repositioning and/or placement of oral airway		
Prebriefing (30 min) • Introduction to simulation, explanation of RCDP, objectives for the training session, introduction of each disciplines’ RCDP coaches, timing of the workshop and an introduction to the mannequin		
Rapid Cycle Deliberate Practice (40) minutes*		
Scenario State	Hard Stops	Soft Stops
I: Hypoxia and bradycardia (5–7 min) Requires nurse to take the patient off of the ventilator and perform bag/mask ventilation ETT displacement Team must use DOPE mnemonic to recognize tube dislodgement and remove ETT	RN: Bag/mask ventilation technique MD: Shares mental model, assigns roles, uses DOPE to recognize tube dislodgement	RN: Recognizes decompensation, calls for help RN/MD/RT: Recognizes DOPE, shares mental model
II: Bag/Mask Ventilation (5–7 min) Team must perform bag/mask ventilation and prepare for intubation	RN: Medication safety closed and directed communication RT: Bagging technique	
III: Preintubation (15 min) Team must draw medications and obtain necessary equipment for intubation	RN: Uses QI bundle checklist to review anticipated medications for intubation. Confirms medications with provider MD/RT: MD and RT review the QI bundle checklist and use the plan generation component to obtain the correct equipment for intubation	RT: Selects appropriate airway equipment RN: Retrieves nasal cannula from the nursing supply cart for apneic oxygenation MD intubator: Assigns a second MD as team leader to ensure completion of the check list elements
IV: Intubation preparation (3–5 min) Team uses NEAR4KIDS QI bundle checklist to prepare for intubation	RT: Initiatives apneic oxygenation and verbalizes it is use and rationale MD: Ensures that all team members have completed tasks before initiation of the checklist and are ready to proceed with the time out MD team lead: Initiates preprocedure time out MD Intubator: Reviews risk assessment and planning (who will intubate, how we they intubate, when will they intubate, and backup plan) MD intubator: Assigns team roles MD team lead: Prompts review of risk assessment and plan generation (correct equipment, monitoring, rescue plan)	MD/RT: Review appropriate liter flow for apneic oxygenations as suggested by NEAR4KIDS RT/RN: Shares mental model
V: Intubation (2 min) Patient is sedated, physician intubates, and RT secures ETT	RN: Sedates the patient with fentanyl, versed and rocuronium using closed loop communication RT: Assess for air leak following securement of ETT	MD: Provides bag/mask ventilation with apneic oxygenation cannula in place MD: Intubates using direct laryngoscopy RT/MD: Provides bag/mask ventilation once ETT in place RT: Secures ETT
Traditional reflective debriefing (40 min)*		
Reactions phase; 5 min, Descriptive phase; 5 min, Analysis phase; 20–30 min	• Learner centered discussion • Facilitators framed take-away messages • Learners discussed barriers to implementation of the bundle, perceptions of use and application of the risk assessment/plan generation, and solutions and opportunities for improvements to promote bundle adherence • Additional team training concepts discussed related to the intubation process; closed loop communication, directed communication, role assignment, shared mental model, and prioritization.	

*The time duration detailed here reflects the time spent on deliberately practicing bundle implementation during RCDP and time spent discussing barriers to implementation during TRD. The remainder of the three-hour workshop was dedicated to additional learning objectives beyond the scope of this article.

DOPE, Dislodgement, Obstruction, Pneumothorax, Equipment; ETT, endotracheal tube.

### Study of the Intervention

Translational simulation encompassed 3 elements: exploration, testing, and embedding. Through exploration and testing, learners actively engaged in the intubation process, and effectively unmasked process limitations, elucidating the rationale for poor adherence to the bundle from the clinician’s perspective. As simulation elucidated how the bundle did not fit into the workflow, iterative changes were adopted and embedded in subsequent training sessions, increasing the simulation-based intervention’s reliability.

### Measures

A yellow checklist form created by NEAR4KIDS outlined three bundle elements; (1) risk factor assessment and plan generation; (2) preprocedure time out; and (3) postprocedural huddle. As defined by NEAR4KIDS registry, the use of the bundle was reported as (1) fully used (all 3 sections of the checklist as described above); (2) partially used (only 1 or 2 sections of the checklist used); or (3) not used. We calculated the compliance rate from TIs with the fully completed checklist divided by all TI encounters for a given period. Apneic oxygenation compliance defined by NEAR4KIDS guidelines was measured as a discrete element. Data were collected by retrospective chart review by our NEAR4KIDS coordinator. Bundle use and occurrence of adverse TIAEs was reported from the network back to our site.^1^ A monthly compliance rate equal to or above 80% was considered adherent by the network.^1^ TIAEs were defined by NEAR4KIDS. TIAE rates were calculated by TI encounters with one or more adverse events divided by all TI encounters.^5^

## ANALYSIS

Medians and interquartile ranges for continuous variables and frequencies and percentages for categorical variables summarized patient demographics, diagnoses, indications for intubation, compliance, and TIAE data. We excluded data during the implementation period. Data were collected in two distinct eras (ie, no repeated patient-level observations) and contingent on expected frequency counts. Wilcoxon rank-sum tests evaluated continuous data. Chi-square tests of independence and Fisher’s exact tests evaluated discrete data. SAS v.9.4 (Cary, N.C.) was used for analysis with statistical significance at the 0.05 threshold. Microsoft Excel 2010 was used for statistical process control data analysis, display, and control chart development. We used the American Society for Quality special-cause rules to examine the control charts and separate common-cause versus a special-cause variation.^15^

## RESULTS

**Fig. 1.**
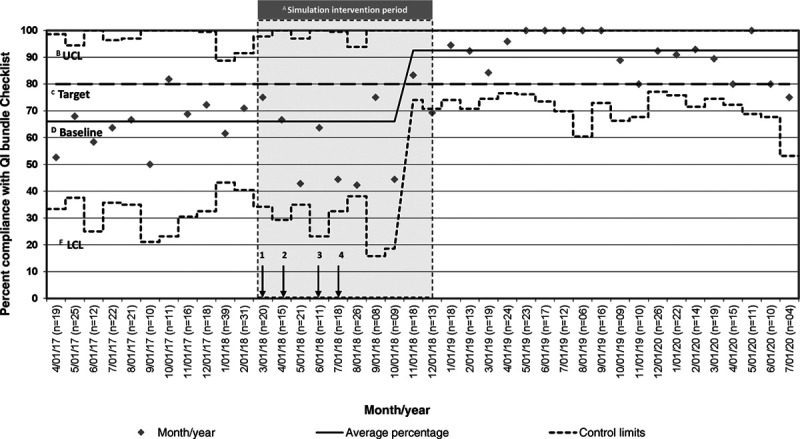
Statistical process control p-chart demonstrating changes to bundle adherence per month. A, Intervention period; B UCL, Upper control limit; C, Compliance goal of equal ≥80%; D, Baseline; E, Lower control limit 1 Role of second physician clarified, 2 Checklist location standardized, 3 Nasal cannula added to the nursing bedside cart, 4 Liter flow for apneic oxygenation placed on video laryngoscope.

Overall, 180 learners, including 19 pediatric critical care medicine fellows, 4 critical care nurse practitioners, 127 nurses, and 30 RTs, participated in the simulation. Bundle compliance and rates of TIAEs were collected on 244 TIs in the 12-month preintervention period and on 158 TIs in the 9-month postintervention period. Patient demographics are in Table 2. Bundle compliance was 66% preintervention and 93.7% postintervention (*P* < 0.001). Statistical process control p-chart demonstrating changes to bundle adherence per month is in Figure 1.^16^ Adherence to apneic oxygenation was 27.9% preintervention simulation and 77.9% postintervention (*P* < 0.001) (Table 3). There was no statistically significant difference in the occurrence of TIAEs. Although the rate of TIAEs increased in the postintervention period, the rate of severe TIAEs decreased by 42% (Table 3). In the 19 months since the completion of translation simulations, bundle compliance remains >90% (Fig. 1). Table 4 summarizes the key barriers to implementation identified and subsequent process improvements made.

**Table 2.** Patient Demographics Presimulation and Postsimulation Intervention

	Preintervention, N = 244	Postintervention, N = 158	*P*
Age (y), Median (25th, 75th)	2.2 (0.5, 11)	2.2 (0.7, 9.9)	0.972
Gender			
Male	132 (54.1%)	58 (36.7%)	0.001
Female	112 (45.9%)	100 (63.3%)	
Weight (kg), Median (25th, 75th)	13.1 (6.4, 35.8)	12.3 (6.8, 35)	0.886
Diagnosis			
Cardiac surgery	0 (0%)	0 (0%)	
Cardiac medical	3 (1.2%)	10 (6.3%)	0.005
Upper airway	17 (7%)	16 (10.1%)	0.260
Lower airway	134 (54.9%)	65 (41.1%)	0.007
Sepsis/shock	29 (11.9%)	19 (12%)	0.966
Neurologic	37 (15.2%)	37 (23.4%)	0.037
Trauma	16 (6.6%)	11 (7%)	0.874
Other	8 (3.3%)	7 (4.4%)	0.552

**Table 3.** Compliance with NEAR4KIDS Airway Safety QI Bundle Checklist, Apneic Oxygenation and Incidence of TIAEs Presimulation and Postsimulation Intervention

	Preintervention, N = 244	Postintervention, N = 158	*P*
NEAR4KIDS airway safety QI bundle checklist compliance	161 (66%)	148 (93.7%)	<0.001
Apneic oxygenation compliance	68 (27.9%)	123 (77.9%)	<0.001
Total encounters with tracheal intubation adverse events	17 (6.9%)	14 (8.8%)	†0.487
Total encounters with nonsevere adverse events	10 (4.90%)	11 (6.9%)	†0.252
Total encounters with severe adverse events	7 (2.8%)	3 (1.9%)	†0.746
Adverse event category*			
**Cardiac arrest (died**)	**0**	**0**	
**Cardiac arrest (survived**)	**1**	**1**	
Mainstem intubation	1	3	
Esophageal intubation (immediate recognition)	3	2	
**Esophageal intubation (delayed recognition**)	**0**	**0**	
**Vomit with aspiration**	**2**	**0**	
Vomit without aspiration	0	1	
**Hypotension needing intervention**	**4**	**2**	
Hypertension needing intervention	0	0	
Epistaxis	0	0	
Gum/dental trauma	1	2	
Lip trauma	2	1	
**Laryngospasm**	**0**	**0**	
**Malignant hyperthermia**	**0**	**0**	
Medication error	0	0	
**Pneumothorax/pneumomediastinum**	**0**	**0**	
Dysrhythmia (including bradycardia < 60 bpm)	1	2	
**Direct airway injury**	0	**1**	
Pain/agitation requiring additional medication and delay in intubation	2	0	

*The number of specific TIAEs are ≥ the number of TI encounters associated with an adverse event as more than one adverse event may occur in a single TI encounter. Severe adverse events are identified in bold.

†*P* are calculated based on TI encounters during which a TIAE or severe TIAE occurred.

**Table 4.** Key Barriers to Bundle Implementation Identified during Simulation and Process Improvements Made

Intubation Process: The intubation process outlined reflects our local culture and practice before simulation.		
▪ NEAR4KIDS QI bundle checklist form is population with patient information, assessment, and plan generation before intubation		
▪ Decision is made to intubate patient		
▪ Physician reviews medications that are needed for intubation with patient’s primary nurse		
▪ Two nurses (primary nurse and resource, or 2 resource nurses) draw medications		
▪ MD discussed with RT what supplies and equipment is needed (ETT size, laryngoscope type and size, airway adjuncts, ventilator settings)		
▪ Primary RT manages patient’s airway (as needed) while resource RT gathers equipment and supplies		
▪ Team assembles and includes 2 physicians (a fellow and attending or 2 fellows), 4 nurses (primary RN, 2 medication RNs, documenting RN), and 2 RTs (primary RT and resource RT)		
▪ Intubating physician and RT check and verify that supplies and equipment are available and functioning		
▪ Intubating physician uses NEAR4KIDS QI bundle checklist based on perceived time availability and urgency of intubation. Second physician is passive and assists laryngoscopist if needed		
▪ Apneic oxygenation applied based on physician discretion		
▪ Patient is intubated and ETT is secured		
	Barriers to Implementation Identified	Improvements Made to Address Barriers
System/cultural barriers	Inconsistent communication between physician and team regarding intubation plan for patient	MD utilizes the QI bundle checklist to review plan with team members
	Poor role assignment and lack of role clarity of second physician	MD intubating assigns a second MD as team leader to ensure completion of all checklist elements
	Due to lack of perceived time and competing priorities, team either aborted bundle application completely or quickly reviewed the elements, skipping components	Use of the QI bundle checklist standardized for all intubations regardless of whether it is planned, urgent, or emergent
	Time out performed while other team members were completing tasks Risk assessment and plan generation reviewed between MD and RT and/or MD and RN in a silo and not consistently with entire team	Physician ensures that all team members complete tasks before initiation of the checklist and are ready to proceed with the time out Intubating physician reviews risk assessment and planning (who will intubate, how we they intubate, when will they intubate, and backup plan) with entire team
	High cognitive load on intubating physician to ensure that all check list elements were completed	Team lead physician stands at the foot of the bed and holds QI bundle checklist form to prompt review of risk assessment and plan generation (correct equipment, monitoring, rescue plan)
Process barriers	QI bundle checklist inconsistently populated with patient information and plan	QI bundle checklist form is populated with patient information, assessment, and plan generation on admission and reviewed before intubation
	QI bundle checklist form not used as a reference for nurses to help anticipate patient risk factors or medications that would be used for intubation	RN uses QI bundle checklist to review anticipated medications for intubation and confirms medications with physician
	QI bundle checklist form not used as a reference for RTs to gather intubation equipment and supplies	RT selects appropriate airway equipment using QI bundle checklist
	Inconsistent use of apneic oxygenation	Team lead physician uses QI bundle checklist to prompt and remind team to apply apneic oxygenation if not initiated earlier RN retrieves nasal cannula from nursing bedside cart for apneic oxygenation
Work environment barriers	Inconsistent location of QI bundle checklist form	Location of QI bundle checklist form within each patient room standardized
	Inconsistent availability of nasal cannula for apneic oxygenation	Nasal cannula stored in nursing bedside cart
	Lack of knowledge of appropriate liter per minute flow for apneic oxygenation by age	Card that details liter per minute flow of oxygen per age for apneic oxygenation (as recommended by NEAR4KIDS) attached to video laryngoscope

ETT, endotracheal tube; MD, medical doctor; RN, registered nurse.

## DISCUSSION

Our study demonstrates that translational simulation improved bundle compliance with the NEAR4KIDS quality initiative. Before implementing this project, our PICUs bundle compliance was well below target, a challenge reported with other centers in the network.^3^ Despite skill decay seen with purely educational interventions and an annual turnover of 30%–40% of our nurses and 25% of our RTs, we have maintained >90% compliance in the 19 months since the intervention was completed without any refresher education. Improved and sustained bundle compliance is unlikely related to the simulation-based education intervention alone.^17^ Translational simulation did more than educate teams on better practice. It engaged teams to explore barriers to compliance and overcome those barriers through iterative process improvements.

The bundle, designed to orchestrate the coordination of tasks and prompt communication patterns in a wide variety of ICUs, does not account for the complex local challenges, processes, and systems issues essential for effective implementation.^6^ Regardless of how the checklist was implemented, to generate the outcome benefits of bundle components, one must consider how human factors augment safety.^6^ Additionally, using simulation as an educational tool alone to hardwire checklist use is not a comprehensive solution to mitigate neither the challenges related to implementation or compliance with QI initiatives nor adherence with safety practices.^17,18^

There is a practical need to imagine how a quality initiative may be integrated into practice. However, solely relying on work as imagined is inadequate and does not fully account for the complexity of actual care delivery.^19^ Translational simulation demonstrated work as done better. It brought clarity to how human behaviors, perceptions, and unit-based culture contributed to complex challenges that teams faced during intubation. For systems to achieve higher levels of safety, it is necessary to identify factors that contribute to noncompliance to create opportunities for improvement. Translational simulation increased bundle compliance through (1) bundle customization using process and system improvements; (2) transforming safety culture; and (3) engaging a multidisciplinary team.

Promoting bundle compliance relies on implementing the bundle in a locally accepted manner that easily integrates into the unit’s microsystem, workflow, and culture.^3^ Local cultural barriers to consistent integration of the bundle included time pressure, demanding patient needs, and competing priorities.^7,20^ Staff felt that reviewing the risk assessment and plan was time-consuming and took away from other patient care needs. Teams only wanted to apply the bundle to non-emergent intubations when there was a perception of adequate time. To save time, staff either aborted bundle use completely or quickly reviewed the elements, skipping components. During the simulation, team members were engaged in medication preparation, answering phone calls, or preparing equipment during the timeout. Redirection demonstrating effective implementation during simulation highlighted how improvement in communication streamlined planning through anticipation of patient needs and reduced redundancy in task load. Standardization of bundle use across all intubations, irrespective of team competency and patient condition, actually maximized workflow efficiency, validating its application as an asset rather than an inconvenience.

Nursing engagement in bundle compliance was reliant on integrating bundle elements into their workflow. The simulation demonstrated that nurses perceived the bundle as a tool that did not apply to their practice. They were often disengaged during bundle review, completing other tasks while the physician and RT reviewed the intubation plan in a silo. During the simulation, nurses referenced the checklist to prepare medications and supplies for intubation based on the bundle risk assessment and mitigation plan. During the reflective debriefing, nurses discussed how the bundle helped them anticipate patient needs, optimizing tasks to improve their workflow.

Simulation highlighted that the laryngoscopist felt it too cumbersome to ensure bundle completion and prepare for intubation, discouraging bundle use. To distribute the intubating physician’s cognitive load, we clarified the role of the second physician. The intubating physician was responsible for reviewing the risk assessment, mitigation plan, and intubation equipment immediately before intubation. Simultaneously, as the team leader, the second physician stood at the foot of the bed, held the checklist form, and ensured that all bundle elements were reviewed by prompting completion of any missed item.

Standardization and consistency in bundle elements in the care environment reduced the cognitive load on caregivers by improving predictability with the space and reducing search and locating actions and errors during emergency events.^21^ Modifications to the work environment included standardizing where the checklist form was located in each patient room, storing a nasal cannula in the nursing bedside cart, and adding a sign on the video laryngoscope that defined liter flow for apneic oxygenation.

Safety culture aims to minimize adverse events in a complex and hazardous work environment.^7^ Components of the bundle, particularly the risk assessment and mitigation planning, were safety strategies easily adopted by frontline staff. During the simulation and anecdotally at the bedside, there was a tendency to conduct a risk assessment and develop a mitigation plan during other complex clinical situations. For example, as the patient decompensated during simulation, staff discussed patient risk factors, anticipated patient needs, preemptively assigned roles, proactively prepared medications, and gathered equipment to support changes under clinical condition. Integrating this strategy into the microsystem triggered a transformation of perspective from “this is the way we always do things” to “how can we do things better?”^7^ Staff began to open their minds to practice change and identify safety issues during high-risk and routine procedures, promoting sustainability in practice.

The simulation was also effective in engaging a multidisciplinary team. Initial NEAR4KIDS education focused on RTs and physicians. Yet, 70% of learners during simulation were nurses, reflecting the demographics of our PICU team. The simulation provided a shared experience that leveled perceptions amongst disciplines, providing nurses with insight into the complex coordination of tasks during intubation. Improved task coordination and team communication empowered nurses to engage in the intubation process and advocate bundle implementation. Multidisciplinary staff engagement and active participation in this quality initiative promoted a transition from being a reactive system where we respond when things go wrong (Safety I) to a proactive culture where we develop preventative solutions to problems before they occur (Safety II).^19^

Reflective Deliberate Practice, a blended technique incorporating RCDP and TRD, was a novel training approach used to target skill acquisition, behavioral transformation, and culture shift. Our centers’ previous experience found that each methodology alone failed to translate to skill mastery or sustained practice change. RCDP, as a debriefing methodology, provided targeted feedback, repetition, and time for teams to practice how to incorporate the bundle into the team’s workflow.^10,22^ TRD promoted practice modification as learners reflected on how the bundle application could impact their practice.^14^

### Challenges and Limitations

In the analysis of our secondary outcomes, we did not find a significant impact on the rate of TIAEs. Although this study noted a significant improvement in compliance, we had an ineffective sample size to detect a reduction in these rare adverse events. Variables that contribute to adverse events in the NEAR4KIDS registry are multifactorial and include patient factors (diagnosis and indication for intubation), provider factors (learner versus advanced trained provider), or practice factors (equipment, medications).^1^ Ability to achieve target compliance without reducing adverse events suggests that latent conditions beyond those previously described contribute to the risk associated with TI. The inability to further characterize adverse events based on the data submitted to the NEAR4KIDS registry limits the ability to establish a comprehensive understanding of modifiable risk factors. Work system interactions and human factors are frequently in play in healthcare. We are likely not accounting for interactions of complex adaptive systems, processes, and human behaviors that contribute to errors.

There are many limitations to this study. The QI educational toolkit developed to address barriers to bundle compliance through bundle customization and multidisciplinary team engagement was not implemented before initiating this project, possibly skewing our low rates of bundle compliance preintervention. Additionally, the NEAR4KIDS education on apneic oxygenation was not distributed before simulations, contributing to our extremely low preintervention compliance.

This report is a single-center study, and therefore, this approach is not validated. Although barriers to the implementation described here are specific and unique to our microsystem and local culture, we believe this approach can be adopted and applied at other centers. The simulated scenario, information elicited during simulations, and opportunities for improvement will be unique to each center’s culture and workflow. Similar translational simulation initiatives need to be implemented in a multicenter study to validate this approach.

## CONCLUDING SUMMARY

Integrating translational simulation is a means to promote a culture of safety and reliability and improve adherence to best practices by addressing barriers in the work system. Ongoing safety work to reduce adverse events related to intubations and other high-risk procedures is warranted as we strive to achieve zero harm.

## ACKNOWLEDGMENTS

We would like to acknowledge the Children’s Healthcare of Atlanta Simulation Center and NEAR4KIDS multicenter airway collaborative network. They have done work to pave the way for this publication.
